# Smoking, COPD, and 3-Nitrotyrosine Levels of Plasma Proteins

**DOI:** 10.1289/ehp.1103745

**Published:** 2011-06-06

**Authors:** Hongjun Jin, Bobbie-Jo Webb-Robertson, Elena S. Peterson, Ruimin Tan, Diana J. Bigelow, Mary Beth Scholand, John R. Hoidal, Joel G. Pounds, Richard C. Zangar

**Affiliations:** 1 Cell Biology and Biochemistry,; 2 Computational Biology and Bioinformatics, and; 3 Scientific Data Management, Pacific Northwest National Laboratory, Richland, Washington, USA; 4 Department of Internal Medicine, Pulmonary Division, University of Utah Health Sciences Center, Salt Lake City, Utah, USA

**Keywords:** cigarette smoke, COPD, ELISA, eNOS, nitrotyrosine, posttranslational modification

## Abstract

Background: Nitric oxide is a physiological regulator of endothelial function and hemodynamics. Oxidized products of nitric oxide can form nitrotyrosine, which is a marker of nitrative stress. Cigarette smoking decreases exhaled nitric oxide, and the underlying mechanism may be important in the cardiovascular toxicity of smoking. Even so, it is unclear if this effect results from decreased nitric oxide production or increased oxidative degradation of nitric oxide to reactive nitrating species. These two processes would be expected to have opposite effects on nitrotyrosine levels, a marker of nitrative stress.

Objective: In this study, we evaluated associations of cigarette smoking and chronic obstructive pulmonary disease (COPD) with nitrotyrosine modifications of specific plasma proteins to gain insight into the processes regulating nitrotyrosine formation.

Methods: A custom antibody microarray platform was developed to analyze the levels of 3-nitrotyrosine modifications on 24 proteins in plasma. In a cross-sectional study, plasma samples from 458 individuals were analyzed.

Results: Average nitrotyrosine levels in plasma proteins were consistently lower in smokers and former smokers than in never smokers but increased in smokers with COPD compared with smokers who had normal lung-function tests.

Conclusions: Smoking is associated with a broad decrease in 3-nitrotyrosine levels of plasma proteins, consistent with an inhibitory effect of cigarette smoke on endothelial nitric oxide production. In contrast, we observed higher nitrotyrosine levels in smokers with COPD than in smokers without COPD. This finding is consistent with increased nitration associated with inflammatory processes. This study provides insight into a mechanism through which smoking could induce endothelial dysfunction and increase the risk of cardiovascular disease.

Cigarette smoking is a major risk factor for cardiovascular and lung diseases such as chronic obstructive pulmonary disease (COPD) and cancer (Iribarren et al. 1999). Similarly, environmental or secondhand exposure to cigarette smoke is a major health concern. For example, in the United States in 2005, it was estimated that environmental smoke exposure caused 3,000 deaths from lung cancer and 46,000 deaths from coronary artery disease (U.S. Department of Health and Human Services 2006), suggesting that cardiovascular disease is the greatest source of smoke-related mortality for low-dose exposure. Increased inflammatory stress and endothelial dysfunction are believed to be the primary mechanisms of smoking-related cardiovascular disease (Munzel et al. 2006).

Endothelial function is largely regulated by nitric oxide, a gas that acts as a key physiological regulator of blood pressure. In endothelial cells, a single nitric oxide synthase (eNOS) is responsible for nitric oxide production (Kubes et al. 1991). This nitric oxide rapidly diffuses into the vascular smooth muscle cells, where it indirectly stimulates relaxation of blood vessels through regulation of contractile proteins via activation of guanylate cyclase (Beckman and Koppenol 1996) or of sarcoplasmic/endoplasmic reticulum calcium ATPase (Adachi et al. 2004). The relaxation of the smooth muscle results in a decrease in vascular resistance and blood pressure and a concomitant increase in blood flow (Loeb and Longnecker 1992; McPherson et al. 1995).

Although nitric oxide is a free radical, it is not a highly reactive one (Beckman and Koppenol 1996; Church and Pryor 1985). An important route of nitric oxide degradation is the rapid reaction with superoxide anion to form the more reactive product, peroxynitrite (ONOO^–^). Peroxynitrite reacts with proteins to form 3-nitrotyrosine (Beckman and Koppenol 1996). Immune cells, including macrophages and neutrophils, simultaneously release nitric oxide and superoxide into phagocytic vacuoles as a means of generating peroxynitrite to kill endocytosed bacteria (Pacher et al. 2007). Other inflammatory cells can also produce reactive chemicals that can result in nitrotyrosine formation, including the peroxidases in activated neutrophils and eosinophils. Thus, protein- associated nitrotyrosine is a characteristic marker of nitrative stress and, commonly, inflammation (Pacher et al. 2007). For example, increased levels of protein-bound nitrotyrosine have been reported in a variety of human diseases with an inflammatory component, including atherosclerosis, myocardial ischemia, multiple sclerosis, Parkinson’s disease, inflammatory bowel disease, amyotrophic lateral sclerosis, diabetes, and many others, as reviewed by Pacher et al. (2007).

Cigarette smoking alters the concentration of exhaled nitric oxide in a time-dependent manner. One minute after smoking cessation, levels of exhaled nitric oxide are increased in smokers (Chambers et al. 1998). Exhaled nitric oxide levels remain statistically elevated at 10 min after smoking, but these values are closer to control levels than to the values obtained at the 1-min time point (Chambers et al. 1998). This transient increase in exhaled nitric oxide probably reflects the fact that cigarette smoke contains up to 300 ppm nitric oxide (Pryor and Stone 1993). Thus, there is a rapid uptake of nitric oxide by the lung during cigarette smoke exposure, followed by a rapid loss after smoking. Two hours after smoking, however, exhaled nitric oxide levels are reduced in smokers relative to controls (Kanazawa et al. 1996). Thus, it seems evident that smoking has a sustained suppressive effect on exhaled nitric oxide levels that is not directly dependent on nitric oxide uptake from cigarette smoke. This sustained reduction in exhaled nitric oxide in smokers may result from inflammatory processes and superoxide-dependent conversion to peroxynitrite (Peluffo et al. 2009). In this conceptual model, the increase in peroxynitrite is predicted to elevate nitrotyrosine levels. One small study (*n* = 5 for the smoking group) found a statistically significant increase in nitrotyrosine levels in blood proteins from smokers compared with nonsmokers, thus supporting the superoxide-degradation model (Peluffo et al. 2009). Studies in animals have also detected an increase in nitrotyrosine in circulating proteins after exposure to cigarette smoke (Kunitomo et al. 2009; Yamaguchi et al. 2007), although it seems unlikely that the doses and dosing regimens used in these studies are reflective of human cigarette smoke exposure.

Even so, there is reason to suspect that mechanisms other than increased production of superoxide and subsequent degradation of nitric oxide may be responsible for the smoking-related suppression of exhaled nitric oxide. There is substantial evidence that smoking can inhibit eNOS activity (Munzel et al. 2006), which may account for the reduction in exhaled nitric oxide in smokers. This concept is further supported by a study in healthy rabbits that suggested that eNOS produces essentially all of the exhaled nitric oxide in healthy animals (Vaughan et al. 2003). Cigarette smoke extracts irreversibly reduce the expression of eNOS in pulmonary artery endothelial cells from pigs (Edirisinghe et al. 2010; Raij et al. 2001; Su et al. 1998). Thus, the lower levels of exhaled nitric oxide in cigarette smokers may reflect a persistent suppression of eNOS, thereby providing insight into the mechanisms by which smoking causes endothelial dysfunction and related cardiovascular diseases. It is noteworthy that inducible NOS (iNOS) is associated with an increase in exhaled nitric oxide in persons with asthma, as reviewed previously (Barnes and Liew 1995), but iNOS is not normally present in the lungs of healthy individuals (Knowles et al. 1990). Thus, the decrease in exhaled nitric oxide observed in healthy smokers cannot be due to suppression of iNOS.

To investigate potential mechanisms by which smoking could affect nitric oxide levels, we used a custom enzyme-linked immunosorbent assay (ELISA) microarray platform to analyze the levels of nitrotyrosine modifications in plasma proteins from 458 individuals. Our findings suggest that cigarette smoking is associated with decreased levels of nitrotyrosine-modified proteins in human blood when compared with controls who had never smoked. In contrast, plasma samples from 193 smokers with COPD had elevated levels of nitrotyrosine-modified proteins, compared with 89 smokers without COPD.

## Materials and Methods

*Study population.* All subjects were recruited and samples were collected under institutional review board–approved protocols at the University of Utah. We complied with all applicable requirements of the federal and state regulations, and obtained informed consent from each subject before the study began. These protocols were reviewed by the Institutional Review Board of the Pacific Northwest National Laboratory before transfer and analysis of the samples. We analyzed plasma samples from 458 participants. Plasma from current smokers, former smokers, and never smokers (total *n* = 410) came from participants in the Genetics of Addiction program (University of Utah Medical School). Former smokers were individuals who had consistently quit smoking for at least 6 months. Never smokers were subjects who had smoked less than one cigarette in their lifetime.

Nonsmoking spouses of participating smokers were recruited to this study as individuals at high risk of exposure to environmental tobacco smoke (*n* = 48). These nonsmoking spouses were defined, in part, by smoking < 100 cigarettes per year. Thus, all 458 subjects were recruited from the same population base (Wasatch County in Utah). The study subjects were recruited from advertisements, medical clinics, and in some cases, through prior study participants. Thus, all plasma samples were collected at the same clinical setting, from the same population base, and using the same protocols, although some nonsmoking subjects were recruited based on their relationship to smokers already in the study. We also independently analyzed data from a subset of the full sample set that included 89 current or former smokers who did not have COPD (non-COPD controls) and 193 smokers with COPD.

To the extent possible (i.e., for 281 of 458 samples), samples were blocked and randomized based on the age, body mass index (BMI), sex, and smoking status (never smokers, former smokers, high risk for environmental exposure, and current smokers) of the subject. Blocking was designed to ensure that the spatial distribution of the samples on the chips and slides would have a random distribution based on smoking status, age, BMI, and sex characteristics. The remaining 177 samples, which could not be fully blocked based on these characteristics, were simply randomized prior to analysis. All subjects underwent spirometry per standardized American Thoracic Society methodology (Wanger et al. 2005). Individuals with COPD were identified based on a forced expiratory volume in 1 sec (FEV_1_) value < 70% of predicted value as adjusted for age, height, and sex and an FEV_1_ to forced vital capacity (FVC) ratio of < 70%. The smokers who were defined as not having COPD (non-COPD) were individuals with FEV_1_ predicted and FVC values in the normal range (> 80%) with normal FEV_1_:FVC ratios. In addition, based on questionnaire results, all subjects in both the COPD and non-COPD groups did not knowingly have asthma, pulmonary fibrosis, or other common lung diseases.

*Processing of plasma samples.* Plasma samples were stored as individual aliquots at –80°C until use. Immediately before analysis, the samples were thawed on ice and centrifuged at 15,000 × *g* for 20 min at 4°C to remove any trace precipitates. The supernatant was diluted 20-fold in 0.1% bovine serum albumin and phosphate-buffered saline containing 100 pg/mL green fluorescent protein, which was used as the antigen in a calibrant sandwich ELISA to identify and normalize any chip-to-chip bias (Daly et al. 2010). The individual capture antibodies [see Supplemental Material, Table 1 (http://dx.doi.org/10.1289/ehp.1103745)] are typically saturated by low nanogram per milliliter concentrations of antigen, and we routinely dilute plasma samples 500-fold to get antigen concentrations in the usable range of the standard curve (Gonzalez et al. 2008a). Thus, the 20-fold dilution of plasma used in this study was expected to saturate the capture antibodies. To test this assumption, four plasma samples were serially diluted and analyzed using the ELISA microarray platform. This test demonstrated that the nitrotyrosine signal was essentially maximal for nearly all of the 24 assays performed here when using a 20-fold dilution (data not shown). Thus, because approximately equal amounts of each capture antibody were printed onto all spots, the signal for nitrotyrosine should reflect the relative amount of nitrotyrosine in a consistent mass of captured antigen and should not be influenced by variations in antigen concentrations.

**Table 1:** Subject characteristics.

All subjects									Subset for COPD analysis				
Characteristic	Total	N	E	Q	S	Total	Non-COPD	COPD
Number (sex)																
Total (% female)		458 (48)		108 (58)		48 (75)		172 (41)		130 (38)		282 (41)		89 (53)		193 (35)
BMI (range, kg/m^2^)																
		29.0 ± 5.9 (15–50)		28.6 ± 6.1 (19–53)		29.8 ± 6.7 (19–50)		28.5 ± 5.4 (15–47)		27.0 ± 5.9 (19–50)		28.6 ± 5.6 (16–48)		29.8 ± 6.6 (19–48)		28.0 ± 4.9 (16–41)
Age																
Years (range)		61.4 ± 8.5		55.9 ± 8.1		61.2 ± 11.0		29.2 ± 5.4		28.2 ± 5.9		63.1 ± 7.1 (43–78)		59.2 ± 7.5 (43–78)		64.8 ± 6.3 (52–76)
Blood pressure																
Diastolic (range, mmHg)		80.8 ± 10.2 (38–118)		79.8 ± 8.9 (58–100)		78.6 ± 10.9 (55–98)		81.8 ± 9.7 (42–100)		81.1 ± 10.7 (38–118)		81.9 ± 9.8		82.9 ± 9.9		95 ± 33
Systolic (range, mmHg)		129.4 ± 15 (98–180)		126.1 ± 15.2 (98–178)		124.9 ± 12.9 (98–168)		132.1 ± 15 (100–180)		130.6 ± 14.7 (102–172)		132 ± 15 (42–118)		132.9 ± 15.1 (65–118)		153 ± 54 (42–110)
Race (%)																
White		399 (99)		111 (100)		NA		176 (98)		113 (99)						
Nonwhite*a*		4 (1)		0 (0)		NA		3 (2)		1 (1)						
FEV_1_																
Percent predicted		NA		NA		NA		68 ± 14		68 ± 15		NA		NA		68 ± 14
Age first smoked																
Years of age (range)		NA		NA		NA		15 ± 4		15 ± 5		15 ± 4		15 ± 5		15 ± 4
coHb																
(range)		NA		3.2 ± 0.9 (0.2–4.8)		NA		3.0 ± 1.1 (0.1–6.4)		6.6 ± 2.7 (0.3–17.3)		4.4 ± 2.5 (0.1–17.3)		5.0 ± 2.0 (1–11)		4.3 ± 2.6 (0.1–17.3)
Time smoking																
Years (range)		NA		0		0		33 ± 11 (2–59)		40 ± 11 (0–64)		36 ± 11 (0–64)		33 ± 12 (2–60)		38 ± 11 (0–64)
Abbreviations: coHb, carboxyhemoglobin; E, high risk for environmental smoke exposure; N, never smoker; NA, data not available; Q, quitter or former smoker; S, current smoker. Data represent mean ± SDs, with the exception of sex and race, which are represented as percentages. **a**In addition to whites, the study included two Hispanics, one African American, and one Pacific Islander. No exclusion or selection criteria were used regarding race. The racial demographics are generally reflective of the population in Wasatch County, Utah, where the subjects were recruited.																

*ELISA microarray analysis.* The ELISA microarray protocols used here are described in detail (Gonzalez et al. 2008b), except that only a single detection antibody was used. In brief, 24 capture antibodies [see Supplemental Material, Table 1 (http://dx.doi.org/10.1289/ehp.1103745)] were printed onto each chip in quadruplicate spots, once in each quadrant. In addition, antibodies for the green fluorescent protein and fiduciary spots were printed in quadruplicate on each chip. Individual chips were incubated with a diluted plasma sample from a single subject, and each sample was analyzed on three chips (see below). A biotinylated antibody (Hycult Biotechnology, Uden, the Netherlands) was used to detect 3-nitrotyrosine in the captured antigens because it showed excellent sensitivity and no detectable cross reactivity with brominated, chlorinated, or unmodified proteins (see Supplemental Material, Table 2). The levels of biotin were amplified using streptavidin conjugated with horseradish peroxidase, and the fluorescent signal intensity was developed using streptavidin conjugated with Alexa 546. After processing, the microarray slides were imaged with a fluorescent laser scanner (ScanArray Express HT, PerkinElmer, Downers Grove, IL) and ScanArray Express software was used to analyze the images and determine the fluorescent intensity of the spots. Data calibration across chips was undertaken using data obtained from a sandwich ELISA for green fluorescent protein, which was spiked into each sample and standard at 100 pg/mL, and using ProMAT Calibrator, a custom, freeware, bioinformatics program that we developed for this purpose (Zangar et al. 2009).

**Table 2:** ELISA microarray ANCOVA on smoking status and 3-nitrotyrosine levels for 24 plasma proteins.

Parametric ANCOVA model (*n* = 458)							Tukey’s adjusted *p*-values										
Plasma proteins	Smoking status	Age	Sex (M > F)	BMI	N > E	N > Q	N > S	E > Q	E > S	Q > S
AGT		5.38E–15		0.935		0.047		0.972		0.274		< 0.001		< 0.001		0.007		0.002		0.858
AmR		1.10E–6		0.553		0.004		0.598		0.004		0.001		< 0.001		0.875		0.999		0.822
CD14		1.33E–8		0.827		0.047		0.742		0.043		< 0.001		< 0.001		0.790		0.586		0.950
CP		1.28E–10		0.943		0.004		0.338		0.026		< 0.001		< 0.001		0.680		0.191		0.546
CRP		2.95E–6		0.910		0.001		0.484		0.015		< 0.001		< 0.001		0.670		0.183		0.536
EGF		3.56E–7		0.748		0.003		0.484		0.018		0.091		0.005		0.526		0.972		0.581
EGFR		9.42E–10		0.782		0.017		0.614		0.031		< 0.001		< 0.001		0.993		0.950		0.979
E-Selct		5.25E–8		0.829		0.162		0.415		0.182		< 0.001		< 0.001		0.463		0.170		0.786
FGF		8.25E–7		0.336		< 0.001		0.946		0.003		0.017		< 0.001		0.428		0.940		0.563
Fibr		2.48E–11		0.816		0.007		0.376		0.024		< 0.001		0.001		0.810		0.277		0.526
HBEGF		5.14E–11		0.951		0.014		0.668		0.054		< 0.001		< 0.001		0.606		0.219		0.711
HGF		1.20E–7		0.987		0.007		0.766		0.002		0.003		< 0.001		0.611		0.979		0.659
ICAM		5.64E–9		0.522		0.005		0.806		0.035		< 0.001		< 0.001		0.933		0.856		0.989
IGF		1.41E–8		0.814		0.012		0.895		0.003		< 0.001		< 0.001		0.959		0.999		0.872
Leptin		1.24E–12		0.819		0.010		0.689		0.263		< 0.001		< 0.001		0.098		0.018		0.722
MMP1		2.75E–9		0.703		0.076		0.589		0.212		< 0.001		< 0.001		0.388		0.096		0.667
MMP2		1.51E–9		0.867		0.161		0.325		0.198		< 0.001		< 0.001		0.276		0.086		0.802
MMP9		3.30E–9		0.817		0.137		0.382		0.166		< 0.001		< 0.001		0.393		0.119		0.740
PDGF		1.01E–14		0.932		0.076		0.736		0.201		< 0.001		< 0.001		0.019		0.013		0.989
RANTES		8.83E–15		0.624		0.017		0.588		0.310		< 0.001		< 0.001		0.016		0.002		0.747
SP-A		7.07E–9		0.735		0.094		0.560		0.056		< 0.001		< 0.001		0.665		0.441		0.940
TGFα		2.16E–11		0.560		0.034		0.433		0.082		< 0.001		< 0.001		0.205		0.089		0.913
TNFα		2.42E–12		1.000		0.016		0.992		0.170		< 0.001		< 0.001		0.225		0.110		0.933
VEGF		4.12E–10		0.870		0.027		0.265		0.125		< 0.001		< 0.001		0.290		0.056		0.626
Abbreviations: AGT, angiotensinogen; AmR, amphiregulin; CD14, cluster factor 14; CP, ceruloplasmin; CRP, C-reactive protein; E, high risk for environmental smoke exposure; EGF, epidermal growth factor; EGFR, EGF receptor; E-selct, E-selectin; FGF, basic fibroblast growth factor; Fibr, fibrinogen; HBEGF heparin-binding EGF; HGF, hepatocyte growth factor; ICAM, intercellular adhesion molecule 1; IGF, insulin-like growth factor 1; MMP, matrix metalloprotease; N, never smokers; PDGF, platelet-derived growth factor A; Q, quitter or former smoker; RANTES, regulated upon activation, normal T-cell expressed, and secreted; S, current smoker; SP-A, surfactant protein A; TGFα, transforming growth factor α; TNFα, tissue necrosis factor α; VEGF, vascular endothelial growth factor.																				

Technical replicates were used at all stages of the ELISA microarray analysis. Each chip had four identical spots for each capture antibody (i.e., four replicates for each ELISA analysis). The median fluorescence value of the four replicate spots was used as the single value for each assay on each chip. In addition, each sample was analyzed on three separate chips, each of which was located on a different slide. Each of the three sets (458 samples/set) of replicate analyses was performed by a different researcher. The nitrotyrosine fluorescent signal intensities were normalized based on the assay and the researcher who did the analysis. The mean value of the three normalized, technical replicates was used as the sole individual value for each assay for all statistical analyses and data summaries presented here.

*Immunoblotting.* Immunoblotting was undertaken essentially as previously described (Zangar et al. 2008), except that no reducing reagent was used. The reducing reagent was omitted based on a previous study that reported that such agents can convert nitrotyrosine residues to aminotyrosine residues (Abello et al. 2009). In tests in our laboratory, the presence of dithiothreitol in the SDS-PAGE (sodium dodecyl sulfate–polyacrylamide gel electrophoresis) loading buffer resulted in no detectable signal in the immunoblots for peroxynitrite-modified albumin, although the same sample produced a strong signal in the absence of reducing agents (data not shown). For the analysis of plasma, individual samples were loaded at 25 µg protein/lane. Levels of 3-nitrotyrosine were determined using an antibody from Hycult Biotechnology.

*Statistics.* An analysis of covariance (ANCOVA) with data from all 458 samples was used to account for all discrete and continuous variables simultaneously. One primary issue for these analyses is that the log-transformed intensity data often did not meet stringent tests of normality (Lilliefors 1967), such that *p*-values to reject the null hypothesis of normality were < 0.001 for all proteins. However, because nonparametric models are very limited in the number of primary factors and covariates that can be included in the model and because the nonparametric ANCOVA showed the same overall significance trend in the primary smoking factor as the full ANCOVA model, only results of the parametric test are presented here. The full ANCOVA parametric analysis testing for all interactions demonstrated no significant interaction effects, and thus the final ANCOVA model given in Table 1 does not include interactions. Finally, a post hoc Tukey’s test (Ott and Longnecker 2001) was used to assess pairwise statistical differences between smoking categories (*p* < 0.05). All basic tests were performed in MatLab® (version R2010a; MathWorks, Natick, MA) using standard functions available through the Statistics Toolbox, and parametric and nonparametric ANCOVA models were implemented in R 2.11.1 using the aov() and T.L2() functions (MathWorks), respectively.

Classification models were developed using partial least squares discriminant analysis (PLS-DA) and evaluated with a receiver operating characteristic (ROC) curve generated from leave-one-out cross-validation. Variable selection was performed using recursive feature elimination (Ott and Longnecker 2001). This analysis works by iteratively removing a single variable from the model based on the area under the curve (AUC). The variable for which removal results in maximizing the AUC is removed, and the process is repeated for the remaining subset to determine the subsequent variable to remove.

## Results

*Subject characteristics.* Of the 458 subjects included in our study, 48% were females, the mean age of all subjects was 61.4 years, and the mean BMI was 29.0 kg/m^2^ (Table 1). The older age of the subjects in this study relative to the general population reflects the age of the COPD population in general, a focus of the original study that these samples came from, and the need to age-match the control subjects. Subjects were not recruited or excluded based on age. Subjects were divided into four groups based on smoking status, including 108 never smokers, 48 individuals at high risk for environmental smoke exposure, 172 former smokers, and 130 current smokers. Blood pressure for all subjects, which was taken at the time of the blood draw, was not associated with smoking status. We also have information on the age of first smoke and for years since quitting for the current and former smokers.

*ELISA microarray analysis of 3-nitrotyrosine proteins.* All 458 samples were analyzed in triplicate using our ELISA microarray platform over a single 24-hr period. Thus, the nitrotyrosine data from the 458 samples for the 24 captured antigens were obtained from 32,976 ELISA analyses. This number does not include the four replicate analyses performed for each ELISA on each chip or the assays for the calibrant protein or other control spots included on each chip. The 24 capture antibodies for the individual antigens analyzed in this study are listed in Supplemental Material, Table 1 (http://dx.doi.org/10.1289/ehp.1103745). We selected these antigens to represent proteins that range from relatively high to low abundance in plasma; that contain tyrosine modifications that we identified by mass spectrometry (unpublished data); that have been reported to be biomarkers for smoking, lung disease, or inflammation; that originate primarily from a single tissue or cell type; and that have suitable antibodies that are commercially available. For example, surfactant protein A (SP-A) was included as a lung-specific protein reported to be increased in the blood in response to chronic smoking (Kida et al. 1997). Similarly, C-reactive protein (CRP) is an inflammatory marker that is increased in the blood of smokers (Vardavas and Panagiotakos 2009).

We initially compared four commercial 3-nitrotyrosine antibodies [see Supplemental Material, Table 2 (http://dx.doi.org/10.1289/ehp.1103745)] in our microarray platform. Of these antibodies, only the nitrotyrosine monoclonal antibody (HM.11) had acceptable sensitivity and specificity (data not shown); that antibody was used for all of the analyses reported here.

**Figure 1:**
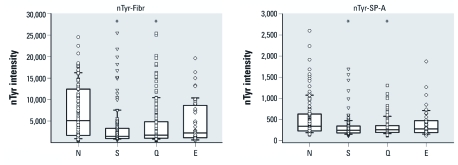
Smoking status and 3-nitrotyrosine (nTyr) levels, as measured by ELISA microarray, for two representative circulating proteins: lung SP-A and fibrinogen (Fibr). Lines within the boxes represent the median values, boxes are 25th and 75th quartiles, and whiskers are the 5th and 95th percentiles of each group. All individual intensity values are shown as data points. E, high risk for environmental smoke exposure; N, never smoked; Q, former smokers (quitters); S, current smokers. *Group significantly different (p < 0.01) compared with N based on a Kruskal–Wallis analysis and a nonparametric Tukey’s post hoc test.

*Smoking and 3-nitrotyrosine levels in plasma proteins.* Average levels of 3-nitrotyrosine in all 24 proteins analyzed with the ELISA microarray platform were decreased in current smokers compared with individuals who had never smoked [statistical results for all assays are provided in Table 2, two representative graphs are shown in Figure 1, and graphs for all 24 assays are shown in Supplemental Material, Figure 1 (http://dx.doi.org/10.1289/ehp.1103745)]. Former smokers also had significantly lower levels than never smokers of 3-nitrotyrosine in 23 of 24 assays at *p* < 0.05 and in all 24 assays at *p* < 0.1. Individuals at higher risk for environmental smoke exposure (as the nonsmoking spouse of a current smoker) had significantly (*p* < 0.05) lower levels of nitrotyrosine in 11 proteins compared with never smokers (Table 2).

We independently validated the ELISA microarray results using immunoblotting. For this validation, plasma samples from seven subjects who had never smoked and from eight smokers were analyzed. The densities associated with the major protein bands in the plasma samples from the never smokers confirmed the results from the ELISA microarray analysis that suggested that smoking broadly reduces nitrotyrosine levels in plasma proteins [Supplemental Material, Figure 2 (http://dx.doi.org/10.1289/ehp.1103745)].

**Figure 2:**
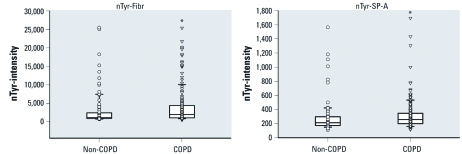
3-Nitrotyrosine (nTyr) levels in representative plasma proteins, lung SP-A and fibrinogen (Fibr), from smokers with COPD and non-COPD smokers. Lines within the boxes represent the median values, boxes represent 25th and 75th quartiles, whiskers represent the 5th and 95th percentiles, and all individual values are shown as data points. *p < 0.01 compared with non-COPD based on a Wilcoxon analysis.

We evaluated the relationship between nitrotyrosine levels and years of smoking, age of first cigarette, blood pressure, and (for former smokers) years since quitting, using a multilinear regression model on the log-transformed intensities (see Supplemental Material, Table 3). These statistical analyses indicate that there is no clear relationship between nitrotyrosine levels and these parameters of smoking behavior tested either as a group (*F*-test) or for each individual variable in the model (*t*-test) in current or former smokers in our study.

**Table 3:** Comparison of 3-nitrotyrosine levels in 24 plasma proteins in smokers with and without COPD.

Plasma proteins	*p*-Value*a*
AGT	0.051
AmR	0.009
CD14	0.034
CP	0.020
CRP	0.022
EGF	0.034
EGFR	0.016
E-Selct	0.049
FGF	0.006
Fibr	0.017
HBEGF	0.014
HGF	0.001
ICAM	0.008
IGF-1	0.011
Leptin	0.010
MMP1	0.025
MMP2	0.078
MMP9	0.032
PDGF	0.012
RANTES	0.009
SP-A	0.070
TGFα	0.012
TNFα	0.005
VEGF	0.015
Pattern	Non-COPD < COPD
Abbreviations: AGT, angiotensinogen; AmR, amphiregulin; CD14, cluster factor 14; CP, ceruloplasmin; CRP, C-reactive protein; EGF, epidermal growth factor; EGFR, EGF receptor; E-selct, E-selectin; FGF, basic fibroblast growth factor; Fibr, fibrinogen; HBEGF heparin-binding EGF; HGF, hepatocyte growth factor; ICAM, intercellular adhesion molecule 1; IGF-1, insulin-like growth factor 1; MMP, matrix metalloprotease; PDGF, platelet-derived growth factor A; RANTES, Regulated upon Activation, Normal T-cell Expressed, aSecreted; SP-A, surfactant protein A; TGFα, transforming growth factor α; TNFα, tissue necrosis factor α; VEGF, vascular endothelial growth factor. Non-COPD are subjects who do not meet criteria of having COPD. **a***p*-Values are based on a Wilcoxon analysis comparing COPD with non-COPD.	

*Nitrotyrosine levels of plasma proteins do not correlate with age or BMI but are associated with sex.* Nitrotyrosine levels were not significantly associated with age or BMI; the associated *p*-values ranged from 0.27 to 1.0 for the ANCOVA model (Table 2). For 18 or 24 proteins, sex was significantly (*p* < 0.05) associated with nitrotyrosine levels. Of additional interest, the ANCOVA included all possible interactions, but none of the interactions were significant (at *p* < 0.05) and therefore were not included in the final model (the results of which are presented in Table 2). In addition, we did not identify any significant differences in nitrotyrosine levels that were clearly based on age or BMI, but our results do indicate a need to consider smoking status and sex when testing for the effects of other variables on nitrotyrosine levels. It is possible that the lack of additional significant interaction effects may be due to limited power based on sample size. Furthermore, interactions between age or BMI and protein nitrotyrosine levels from our study population, which is relatively aged compared with the general population, may not be applicable to comparisons across a more representative set of individuals

*COPD and 3-nitrotyrosine levels in plasma proteins.* We also analyzed the nitrotyrosine levels based on COPD status, which was a key variable in the original design of the Lung Health Study from which some of the samples were obtained. The characteristics (i.e., age, BMI, and sex) of this set of subjects are shown in Table 1. Because COPD is primarily a smoking-related disease, there were insufficient numbers to estimate associations with COPD in individuals who had never smoked. Therefore, we compared sample data from 282 subjects, including 193 smokers with COPD and 89 smokers without COPD (non-COPD). None of the covariates we considered (i.e., age, sex, or BMI) were significant predictors of COPD (data not shown). However, based on a nonparametric Kruskal–Wallis test, smokers with COPD had significantly higher average levels of nitrotyrosine in 21 of 24 plasma proteins than did smokers without COPD (Table 3, Figure 2) [see Supplemental Material, Figure 4 (http://dx.doi.org/10.1289/ehp.1103745)]. The type 2 error (®) under an observed type 1 error (〈) of 0.05, pooled estimate of variance, and the true mean difference estimated from the data showed strong power in these predictions (based on a two-sample test) (Ott and Longnecker 2001). The value of ® ranged from < 1.0E–20 to 2.9E–4. If the observed type 1 error based on a two-sample *t*-test is used instead of a constant 0.05, the values of ® do not change dramatically and range from 9.9E–16 to 1.2E–4.

We also evaluated the relationship between predicted FEV_1_ and nitrotyrosine levels in subjects with COPD, which were the only subjects in this study for which lung function data were available. This analysis indicated that there was no significant relationship between the nitrotyrosine levels in individual proteins and predicted FEV_1_, such that the slopes ranged from –0.002 to 0.002 (*p*-values from 0.22 to 0.97). Thus, we conclude that nitrotyrosine levels in specific proteins were not associated with predicted FEV_1_ in study participants with COPD.

To determine how well the nitrotyrosine-modified proteins could distinguish between individuals with and without COPD, PLS-DA models were performed using data from 133 smokers with and without COPD. (PLS-DA requires complete data and therefore of the 282 individuals in the COPD subset there were 133 with complete clinical data.) The model was then tested on an independent sample set including 55 smokers who were not part of the training sample set. Only the results from this 55-sample test set are presented here. The proportion of subjects with COPD was reasonably consistent between the training and test sets (65% and 75%, respectively). When using only the data available from the nitrotyrosine protein assays, a maximum classification accuracy of ~ 77% in the independent sample set distinguished smokers with and without COPD using the values for nitrated ceruloplasmin (CP), matrix metalloprotease 1, and transforming growth factor alpha (TGF〈). The nitrotyrosine data from other proteins were highly correlated across individuals, such that other combinations of proteins also produced similar classification accuracy (data not shown). A more favorable classification could be achieved by combining clinical measures with the nitrotyrosine data. In these analyses, age, BMI, blood pressure, years smoking, and carboxyhemoglobin (coHb) levels were also evaluated. The final classification model was derived from an iterative variable selection routine, which found that four variables (age, BMI, nitrated CP, and nitrated SP-A) could classify non-COPD from COPD with an accuracy of ~ 86% in the independent sample set. This classification accuracy is significantly better than any single variable, as demonstrated by ROC curves [see Supplemental Material, Figure 5 (http://dx.doi.org/10.1289/ehp.1103745)], which produced AUC values that were between 0.55 and 0.69 for the four individual variables but was 0.81 for the combined variables.

## Discussion

Smoking results in a sustained reduction in exhaled nitric oxide (Kanazawa et al. 1996), which may be related to increased inflammation or inhibition of eNOS. We measured nitrotyrosine levels in plasma proteins in an effort to distinguish between smoking-associated decreases in nitric oxide production and increased nitric oxide degradation that could be related to inflammation and the associated oxidative stress. Nitrative stress is believed to have a role in the etiology of COPD, a smoking-related disease associated with chronic pulmonary inflammation (Ricciardolo et al. 2005). Therefore, we also compared plasma samples from smokers with and without COPD to determine if there was an association between persistent pulmonary inflammation and circulating levels of nitrotyrosine in specific proteins.

This study is distinct from prior studies on nitrotyrosine, which commonly measured total levels of protein-bound nitrotyrosine. Conceivably, the total protein nitrotyrosine levels found in plasma or serum could predominantly represent levels in a single, highly abundant protein, such as albumin, or broad changes in all circulating proteins. A recent review (Pacher et al. 2007) identified > 60 proteins reported to have altered function after tyrosine nitration. These proteins include SP-A and fibrinogen, two proteins analyzed here for nitrotyrosine modifications.

Overall, our study provides a uniquely broad perspective on the nitration levels of individual proteins. We observed that smokers had lower average nitrotyrosine levels in 24 circulating proteins than did nonsmokers or former smokers. In contrast, smokers with COPD had higher average nitrotyrosine modifications in many proteins relative to smokers without COPD. Although smoking is known to stimulate a chronic, low-level pulmonary inflammatory response, our data suggest that any nitrative stress associated with smoking-induced inflammation may be insignificant compared with the suppressive effect of cigarette smoke on nitric oxide production and the subsequent formation of nitrative species. A study examining nitrotyrosine metabolites in urine found that smoking did not alter levels of these metabolites, suggesting that nitrotyrosine degradation pathways are not significantly altered in smokers (Ohshima et al. 1990). Overall, the broad suppression we observed in nitrotyrosine levels in plasma proteins is consistent with the postulate that smoking suppresses endothelial nitric oxide production, which would be expected to decrease nitrotyrosine levels in all circulating proteins to a similar degree. In contrast, the fact that smokers with COPD had higher nitrotyrosine levels than smokers without COPD is consistent with a general inflammatory response that results in increased nitrative stress.

For our ELISA microarray analyses, we used a modest 20-fold dilution of plasma that we believed would essentially saturate the capture antibodies for most of the targeted antigens. Thus, the observed differences in nitrotyrosine levels likely represent relative differences based on a fixed amount of captured protein and generally should not be influenced by variable antigen concentrations in the plasma. In this regard, both SP-A and CRP have been reported to be increased in the blood of smokers. Thus, the fact that we observed lower nitrotyrosine levels in these plasma proteins obtained from smokers strongly supports our conclusion that we are measuring relative changes in the extent of nitrotyrosine modification rather than absolute changes in protein concentrations. This concept is especially relevant for SP-A, which is a lung protein believed to leak into the bloodstream at increased rates during pulmonary inflammation. Because of the normal location of SP-A on the lung surface, SP-A should be directly exposed to cigarette smoke constituents, including nitric oxide, but the association between smoking and nitrotyrosine modification of this protein was essentially indistinguishable from associations with nitrotyrosine levels of any of the other 23 proteins we examined. Because smoking status had similar associations with nitrotyrosine modification of all 24 proteins we assayed, we speculate that protein nitration in the blood may dominate over nitration that occurs in the tissues or cells from which the proteins originated.

One limitation of cross-sectional studies such as the one conducted here is that temporal changes within individuals and temporal relations between exposures and outcomes cannot be evaluated. In addition, the joint effects of COPD and smoking on protein nitrotyrosine levels cannot be adequately assessed in the current study. There may also be other confounding variables that contribute to differences in nitrotyrosine levels in individuals, including environmental exposure to chemicals, drugs, nontobacco aerosols, genetic factors, etc., that were not evaluated in this study.

Although the former smokers in this study had quit smoking from 1 to 35 years prior to the blood sampling, we did not observe a significant correlation between the time since quitting and the levels of nitrotyrosine in the blood [see Supplemental Material, Figure 3 (http://dx.doi.org/10.1289/ehp.1103745)]. Previous studies demonstrated that smoking has irreversible effects on genetic mutations and gene expression profiles in bronchial epithelial cells (Beane et al. 2007; Mao et al. 1997). These changes are detectable up to 20 years after smoking cessation and are believed to have a role in the sustained effects of cigarette smoke exposure on lung cancer risk. Our findings suggest that smoking may have irreversible effects on endothelial cell function and, in particular, eNOS activity.

## Supplemental Material

(72 KB) PDF
