# Supplementary material for: Smoking, COPD, and 3-Nitrotyrosine Levels of Plasma Proteins
Source: Environ Health Perspect. 2011 Jun 6;119(9):1314–20. doi: 10.1289/ehp.1103745 (PMC3230408; doi:10.1289/ehp.1103745)
Supplement: (72 KB) PDF [file ehp.1103745.s001.pdf]

Supplementary Materials

Smoking, COPD and 3-Nitrotyrosine Levels of Plasma Proteins

Hongjun Jin[1], Bobbie-Jo Webb-Robertson[2], Elena S. Peterson[3], Ruimin Tan[1], Diana J. Bigelow[1], Mary Beth Scholand[4], John R. Hoidal[4], Joel G. Pounds[1] and Richard C. Zangar[1]*

[1]Cell Biology and Biochemistry, [2]Computational Biology & Bioinformatics, [3]Scientific Data Management, Division of Fundamental & Computational Sciences, Pacific Northwest National Laboratory, Richland WA USA; and [4]Department of Internal Medicine, Pulmonary Division, University of Utah Health Sciences Center, Salt Lake City, Utah, USA

***Address correspondence to Richard Zangar, Cell Biology and Biochemistry, 790 Sixth Street, J4-02, PNNL, Richland WA, USA.

Tel: (509) 371-7301.

Fax: (509) 371-7304.

Email: richard.zangar@pnl.gov.

Supplementary Material Contents

Supplementary Material Table 1. Selected candidate plasma biomarkers and related references for smoking studies. Page 3.
Supplementary Material Table 2. 3-nitrotyrosine antibodies that were tested in the ELISA microarray platform.. Page 4.

Supplementary Material Table 3. Relationship between nitrotyrosine levels and characteristics of smokers. . Page 5.

Supplementary Material Figure 1. Smoking status and 3-nitrotyrosine levels, as measured by ELISA microarray, for 24 circulating proteins. Page 6.

Supplementary Material Figure 2. Immunoblot analysis the levels of 3-nitrotyrosine in plasma proteins. Page 7.

Supplementary Material Figure 3. Lack of changes in nitrotyrosine levels over time in past smokers. Page 8.

Supplementary Material Figure 4. 3-nitrotyrosine levels in 24 plasma proteins from smokers with COPD and smokers without COPD. Page 9.

Supplementary Material Figure 5. A receiver operator characteristic (ROC) curve demonstrating the classification potential of integrating nitrotyrosine protein assay and clinical data. Page 10.

Supplementary Material Table 1. Selected candidate plasma biomarkers and related references for smoking studies.

Capture antibodies	Abbrev.	Catalog # and Supplier	References
angiotensinogen	AGT	MAB3156 [1]	(Mollsten et al. 2008;Seno et al. 1997)
amphiregulin	AmR	MAB262 [1]	(Du et al. 2005;Lemjabbar et al. 2003)
CD14	CD14	MAB3833 [1]	(Choudhry et al. 2005;Droemann et al. 2005;Zhou et al. 2009)
ceruloplasmin	CP	sc-69767 [2]	(Bridges et al. 1986;Galdston et al. 1984;Pignatelli et al. 2001)
C-reaction protein	CRP	MAB17071[1]	(Fujimori et al. 1993;Levitzky et al. 2008)
epidermal growth factor (EGF)	EGF	DY236 kit [1]	(Leikauf et al. 2002;Richter et al. 2002;Willey et al. 1987)
EGF receptor (extracellular domain)	EGFR	AF-231 [1]	(Leikauf et al. 2002;Richter et al. 2002;Schneider et al. 1999)
E-selectin	Esel	AF-724 [1]	(Chen et al. 2004;Churg et al. 2003;Gonzalez et al. 1996)
basic fibroblast growth factor	FGFb	MAB233 [1]	(Morimoto et al. 1999;Noordhoek et al. 2003)
fibrinogen	Fibr	ID6-250310 [3]	(Kourisky and Happert 1964;Pignatelli et al. 2001;Weitz et al. 1992)
heparin-binding epidermal growth factor	HBEGF	AF-292 [1]	(Basbaum et al. 2002;Newland and Richter 2008;Richter et al. 2002)
hepatocyte growth factor	HGF	MAB694 [1]	(Gunella et al. 2006;Stabile et al. 2002)
intracellular adhesion molecular 1	ICAM	MAB720 [1]	(Floreani et al. 2003;Gonzalez et al. 1996;Li et al. 2008)
insulin-like growth factor 1	IGF-1	MAB291 [1]	(Cook et al. 1993;Kim et al. 2007;Liu et al. 2003)
leptin	Leptin	MAB398 [1]	(Chen et al. 2006;Chen et al. 2008;Hansel et al. 2009;Vernooy et al. 2009)
matrix metalloprotease 1	MMP1	AF901 [1]	(Cawston et al. 2001;DeMeo et al. 2007;Klinchid et al. 2009)
matrix metalloprotease 2	MMP2	AF902 [1]	(Kader et al. 2007;Zhang et al. 2009)
matrix metalloprotease 9	MMP9	AF911 [1]	(Nakamaru et al. 2009;Sampsonas et al. 2007;Zayas et al. 2004)
platelet-derived growth factor AA	PDGF	MAB221 [1]	(Churg et al. 2006;Francus et al. 1992;Rom et al. 1991)
RANTES	RANTES	MAB678 [1]	(Bracke et al. 2007;Brozyna et al. 2009;Ma et al. 2005;Oltmanns et al. 2005)
lung surfactant protein A	SP-A	LS-C17957 [4]	(Behera et al. 2005;Honda et al. 1996;Kida et al. 1997;Robin et al. 2002)
Transforming growth factor alpha	TGFa	AF-239 [1]	(Huang et al. 2008;Klein-Szanto et al. 1992;Vlahos et al. 2006)
tumor necrosis factor alpha	TNFa	MAB610 [1]	(Churg et al. 2002;Shao et al. 2004;Wright et al. 2007)
vascular endothelial growth factor	VEGF	AF-293 [1]	(Marwick et al. 2006;Plataki et al. 2006;Suzuki et al. 2008)

Commercial supplier:
[1]R&D Systems; Minneapolis, MN, USA.
[2]Santa Cruz Biotechnology, Inc, Santa Cruz, CA, USA.
[3]ABBiotek, San Diego, CA, USA
[4]Lifespan Biosciences, Seattle, WA, USA.

Supplementary Material Table 2. 3-nitrotyrosine antibodies that were tested in the ELISA microarray platform.

Detection antibodies for 3-nitrotyrosine	Catalog number	Antibodies Resources
1	HM5002	Biotinylated monoclonal antibody to 3-nitrotyrosine (HM11) Hycult Biotechnology b.v., from Netherlands
2	A-21285	Rabbit anti-3-nitrotyrosine polyclonal Antibody from Molecular Probes
3	K97520G	Goat anti-3-nitrotyrosine polyclonal antibody from Meridian Life Science, Inc, Saco, ME
4	Ab27648	Goat anti 3-nitrotyrosine polyclonal from Abcam, Cambridge, MA

Supplementary Material Table 3. Relationship between nitrotyrosine levels and characteristics of smokers. ELISA microarray analysis on the linear relationship between 3-nitrotyrosine levels for 24 plasma proteins against five clinical parameters that are associated with smoking, including years since quitting smoking, age of first cigarette, total number of years smoking, diastolic and systolic blood pressure (BP). The regression model used in the statistical analyses tests the hypothesis that all coefficients are equal to 0 (i.e., $H_O : \beta_1 = \beta_2 = \beta_3 = \beta_4 = \beta_5 = 0$). This analysis indicates that there is very poor association between the measured log-transformed nitrotyrosine signal intensity and the independent variables included in the model.

	Years Quit	Age first Cigarette	Years Smoking	BP Diastolic	BP Systolic
Protein	β_1	β_2	β_3	β_4	β_5
AGT	-0.0016	0.0120	-0.0071	-0.0010	-0.0012
AmR	0.0039	0.0078	0.0007	0.0045	-0.0017
CD14	-0.0017	0.0072	-0.0032	0.0021	-0.0007
CP	0.0015	0.0139	-0.0034	-0.0001	-0.0003
CRP	0.0008	0.0045	-0.0007	0.0022	0.0001
EGF	0.0037	0.0072	-0.0013	0.0052	-0.0011
EGFR	0.0014	0.0148	-0.0062	-0.0026	0.0010
E-Selct	0.0005	0.0146	-0.0072	-0.0031	-0.0007
FGF	0.0004	0.0039	-0.0022	0.0013	0.0006
Fibr	-0.0011	0.0087	-0.0060	-0.0014	-0.0005
HBEGF	0.0016	0.0076	-0.0046	0.0007	-0.0010
HGF	0.0030	0.0071	0.0009	0.0048	-0.0019
ICAM	0.0034	0.0053	-0.0008	0.0033	0.0001
IGF 1	0.0026	0.0049	-0.0009	0.0045	-0.0022
Leptin	0.0027	0.0083	-0.0028	0.0016	-0.0016
MMP1	-0.0010	0.0124	-0.0057	-0.0001	-0.0013
MMP2	-0.0005	0.0129	-0.0072	-0.0018	0.0003
MMP9	-0.0009	0.0168	-0.0080	-0.0025	-0.0002
PDGF	0.0001	0.0095	-0.0043	0.0007	-0.0005
RANTES	0.0009	0.0117	-0.0058	-0.0013	0.0003
SP-A	-0.0010	0.0055	-0.0039	0.0004	-0.0001
TGFa	0.0004	0.0160	-0.0069	-0.0023	0.0012
TNFa	0.0017	0.0092	-0.0025	0.0025	-0.0006
VEGF	-0.0024	0.0172	-0.0101	-0.0042	0.0016

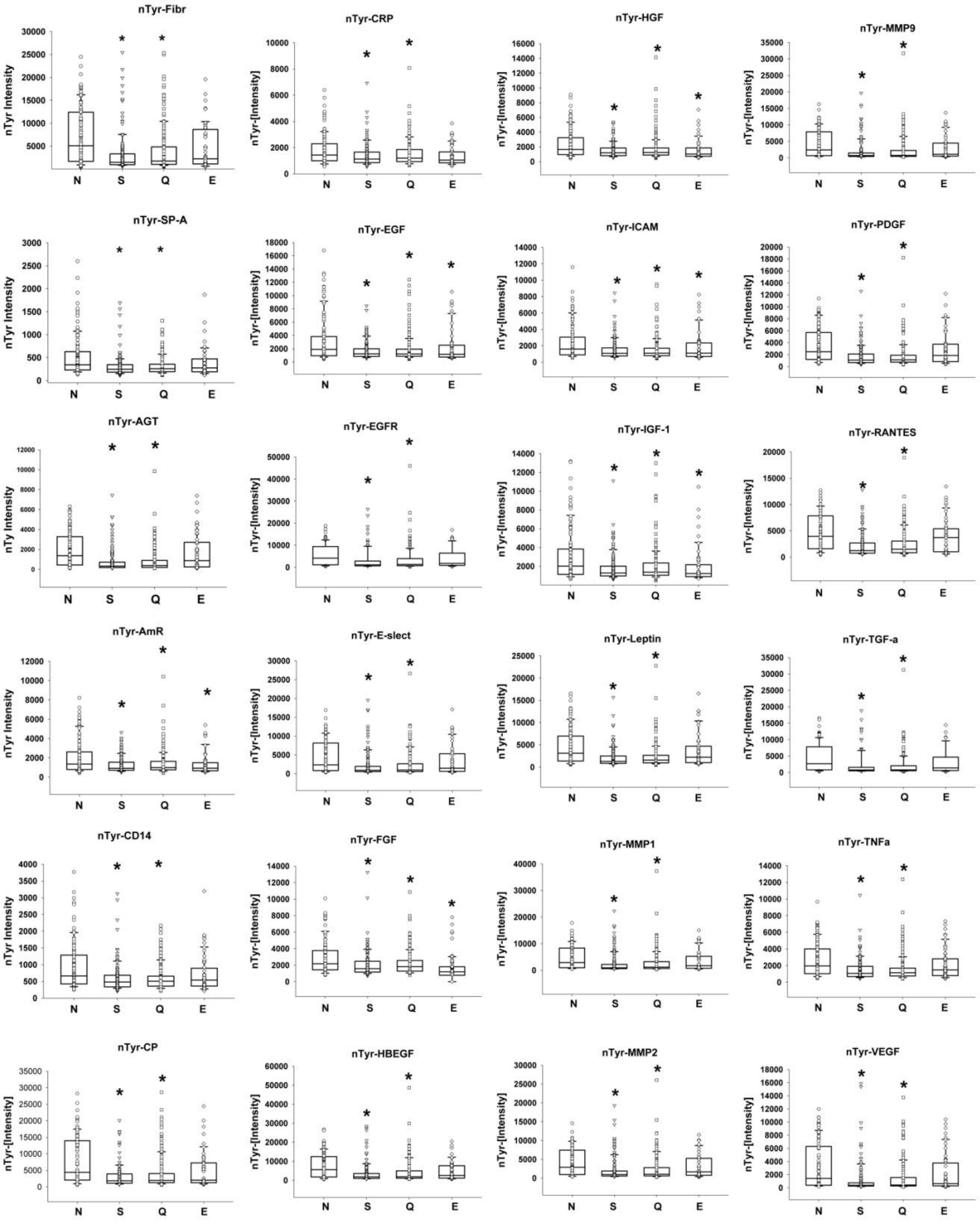

Supplementary Material Figure 1. Smoking status and 3-nitrotyrosine levels, as measured by ELISA microarray, for 24 circulating proteins. The horizontal line within the box represent the median values, the box edges represent the 25th and 75th quartiles, and whiskers represent for the 5th and 95th percentiles of each group. Individual intensity values are shown as individual symbols. Key: current smokers (S), former smokers (Q), never smoked (N), or high risk for environmental smoke exposure (E)* Indicated group value is significantly different ($p < 0.01$) from N group, based on a Kruskal Wallis analsis and a nonparametric Tukey's post-hoc test. (E, diamond). Stars (*) represent $p<0.01$ when S or Q are compared to N based on a Kruskal Wallis analysis and a nonparametric Tukey's post-hoc test.

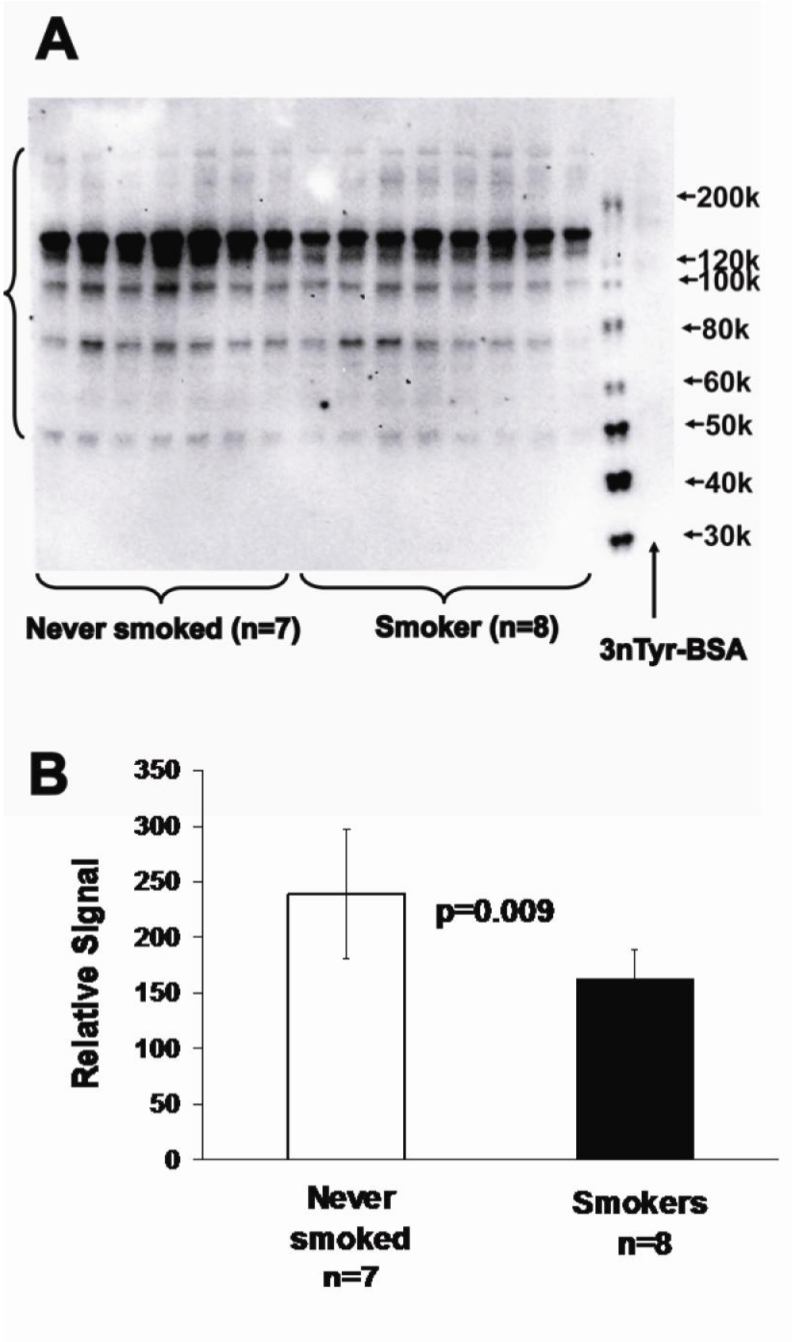

Supplementary Material Figure 2. Immunoblot analysis of the 3-nitrotyrosine levels of plasma proteins. Plasma samples from individuals who had never smoked and or were current smokers were randomly selected and compared. **A**. Image of the immunoblot from ~30 to 250 kDa. **B**. Results from densitometric analysis spanning the bracketed area of the immunoblot that is shown in panel A. Columns and crossbars represent the median density and standard error, respectively, of all the individual lanes per group. The p value (0.009) shows the statistical difference between the two groups, and was determined using a t-test.

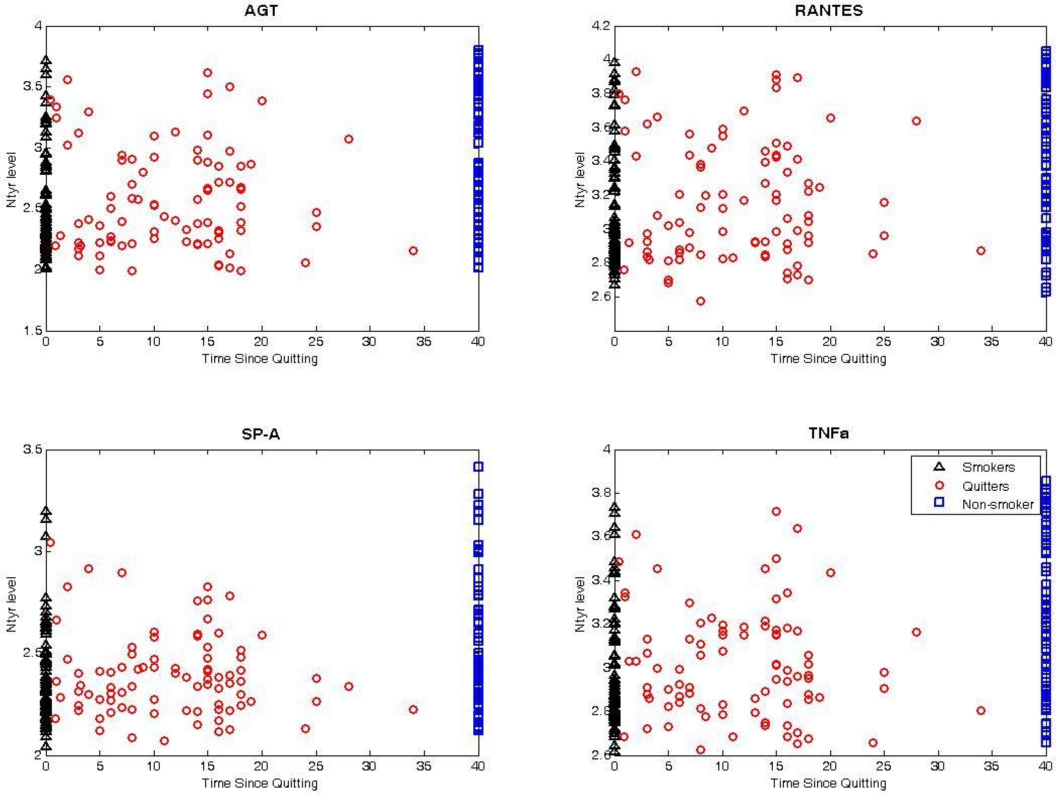

Supplementary Material Figure 3. Nitrotyrosine levels are not associated with time-since-quitting in past smokers. ELISA microarray analysis of current nitrotyrosine levels for select, representative, plasma proteins in past smokers (red diamonds), graphed against the years since quitting. Data from current smokers (black triangles, on left side of graphs) and never smokers (blue squares, on right side of graphs) are also shown. The nitrotyrosine signal intensity (Y-axis) is graphed as the log-transformed data.

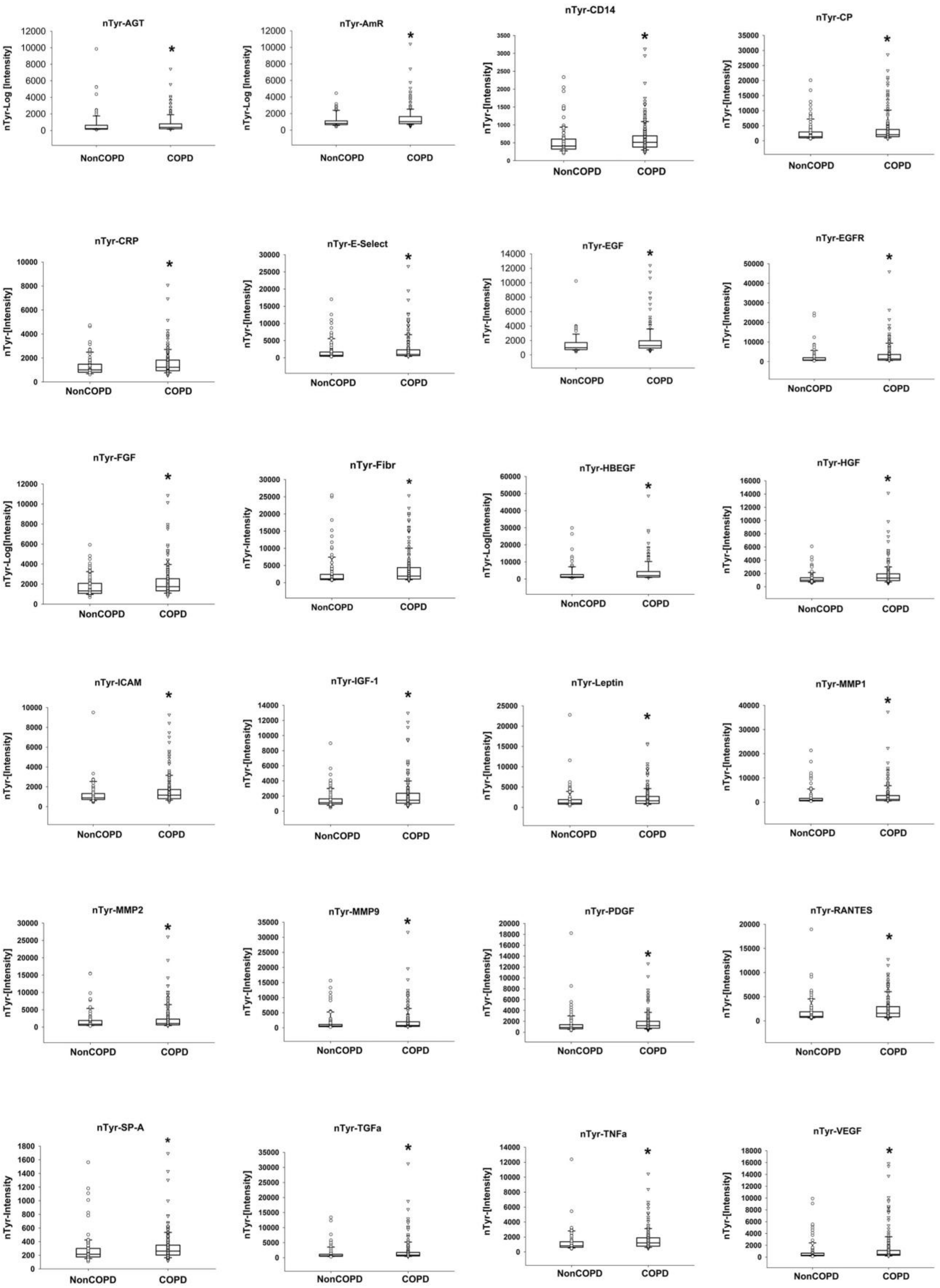

Supplementary Material Figure 4. 3-nitrotyrosine levels in 24 plasma proteins from smokers with chronic obstructive pulmonary disease (COPD) and smokers without COPD (nonCOPD). The horizontal bars within the boxes represent the median values, the boxes extend to the 25th and 75th percentiles, and the whiskers extend to the 5th and 95th percentiles, and all individual values are shown as stated symbols. * The COPD group is significantly different (p<0.01) from NonCOPD group based on a Wilcoxon test.

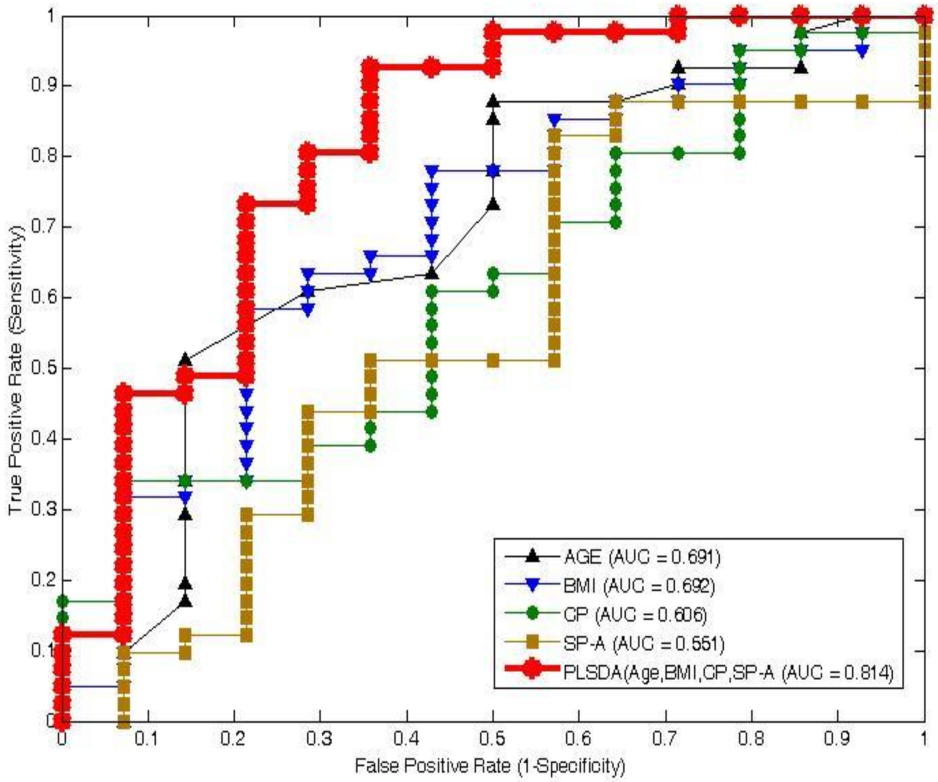

Supplementary Material Figure 5. A receiver operator characteristic (ROC) curve demonstrating the classification potential of integrating nitrotyrosine protein assay and clinical data. Key: AUC. Area under the ROC curve; BMI, body mass index; CP, ceruloplasmin; SP-A, surfactant protein A; PLSDA, partial least squares discriminant analysis.

Reference List

Abello N, Kerstjens HA, Postma DS, Bischoff R. 2009. Protein tyrosine nitration: selectivity, physicochemical and biological consequences, denitration, and proteomics methods for the identification of tyrosine-nitrated proteins. J Proteome Res 8: 3222-3238.

Basbaum C, Li D, Gensch E, Gallup M, Lemjabbar H. 2002. Mechanisms by which gram-positive bacteria and tobacco smoke stimulate mucin induction through the epidermal growth factor receptor (EGFR). Novartis Found Symp 248: 171-176.

Behera D, Balamugesh T, Venkateswarlu D, Gupta A, Majumdar S. 2005. Serum surfactant protein-A levels in chronic bronchitis and its relation to smoking. Indian J Chest Dis Allied Sci 47: 13-17.

Bracke KR, D'hulst AI, Maes T, Demedts IK, Moerloose KB, Kuziel WA, et al. 2007. Cigarette smoke-induced pulmonary inflammation, but not airway remodelling, is attenuated in chemokine receptor 5-deficient mice. Clin Exp Allergy 37: 1467-1479.

Bridges RB, Wyatt RJ, Rehm SR. 1986. Effects of smoking on inflammatory mediators and their relationship to pulmonary dysfunction. Eur J Respir Dis Suppl 146: 145-152.

Brozyna S, Ahern J, Hodge G, Nairn J, Holmes M, Reynolds PN, et al. 2009. Chemotactic mediators of Th1 T-cell trafficking in smokers and COPD patients. COPD 6: 4-16.

Cawston T, Carrere S, Catterall J, Duggleby R, Elliott S, Shingleton B, et al. 2001. Matrix metalloproteinases and TIMPs: properties and implications for the treatment of chronic obstructive pulmonary disease. Novartis Found Symp 234: 205-218.

Chen H, Hansen MJ, Jones JE, Vlahos R, Anderson GP, Morris MJ. 2008. Long-term cigarette smoke exposure increases uncoupling protein expression but reduces energy intake. Brain Res 1228: 81-88.

Chen H, Hansen MJ, Jones JE, Vlahos R, Bozinovski S, Anderson GP, et al. 2006. Cigarette smoke exposure reprograms the hypothalamic neuropeptide Y axis to promote weight loss. Am J Respir Crit Care Med 173: 1248-1254.

Chen HW, Chien ML, Chaung YH, Lii CK, Wang TS. 2004. Extracts from cigarette smoke induce DNA damage and cell adhesion molecule expression through different pathways. Chem Biol Interact 150: 233-241.

Choudhry S, Avila PC, Nazario S, Ung N, Kho J, Rodriguez-Santana JR, et al. 2005. CD14 tobacco gene-environment interaction modifies asthma severity and immunoglobulin E levels in Latinos with asthma. Am J Respir Crit Care Med 172: 173-182.

Churg A, Dai J, Tai H, Xie C, Wright JL. 2002. Tumor necrosis factor-alpha is central to acute cigarette smoke-induced inflammation and connective tissue breakdown. Am J Respir Crit Care Med 166: 849-854.

Churg A, Tai H, Coulthard T, Wang R, Wright JL. 2006. Cigarette smoke drives small airway remodeling by induction of growth factors in the airway wall. Am J Respir Crit Care Med 174: 1327-1334.

Churg A, Wang RD, Tai H, Wang X, Xie C, Dai J, et al. 2003. Macrophage metalloelastase mediates acute cigarette smoke-induced inflammation via tumor necrosis factor-alpha release. Am J Respir Crit Care Med 167: 1083-1089.

Cook RM, Miller YE, Bunn PA, Jr. 1993. Small cell lung cancer: etiology, biology, clinical features, staging, and treatment. Curr Probl Cancer 17: 69-141.

Davis MT, Preston JF. 1981. A simple modified carbodiimide method for conjugation of small-molecular-weight compounds to immunoglobulin G with minimal protein crosslinking. Anal Biochem 116: 402-407.

DeMeo DL, Hersh CP, Hoffman EA, Litonjua AA, Lazarus R, Sparrow D, et al. 2007. Genetic determinants of emphysema distribution in the national emphysema treatment trial. Am J Respir Crit Care Med 176: 42-48.

Droemann D, Goldmann T, Tiedje T, Zabel P, Dalhoff K, Schaaf B. 2005. Toll-like receptor 2 expression is decreased on alveolar macrophages in cigarette smokers and COPD patients. Respir Res 6: 68.

Du B, Altorki NK, Kopelovich L, Subbaramaiah K, Dannenberg AJ. 2005. Tobacco smoke stimulates the transcription of amphiregulin in human oral epithelial cells: evidence of a cyclic AMP-responsive element binding protein-dependent mechanism. Cancer Res 65: 5982-5988.

Floreani AA, Wyatt TA, Stoner J, Sanderson SD, Thompson EG, len-Gipson D, et al. 2003. Smoke and C5a induce airway epithelial intercellular adhesion molecule-1 and cell adhesion. Am J Respir Cell Mol Biol 29: 472-482.

Francus T, Romano PM, Manzo G, Fonacier L, Arango N, Szabo P. 1992. IL-1, IL-6, and PDGF mRNA expression in alveolar cells following stimulation with a tobacco-derived antigen. Cell Immunol 145: 156-174.

Fujimori K, Suzuki E, Arakawa M. 1993. [A case of chronic persistent cough caused by gastroesophageal reflux]. Nihon Kyobu Shikkan Gakkai Zasshi 31: 1303-1307.

Galdston M, Levytska V, Schwartz MS, Magnusson B. 1984. Ceruloplasmin. Increased serum concentration and impaired antioxidant activity in cigarette smokers, and ability to prevent suppression of elastase inhibitory capacity of alpha 1-proteinase inhibitor. Am Rev Respir Dis 129: 258-263.

Gonzalez S, Hards J, van ES, Hogg JC. 1996. The expression of adhesion molecules in cigarette smoke-induced airways obstruction. Eur Respir J 9: 1995-2001.

Gunella G, Bardelli C, Amoruso A, Viano I, Balbo P, Brunelleschi S. 2006. Macrophage-stimulating protein differently affects human alveolar macrophages from smoker and non-smoker patients: evaluation of respiratory burst, cytokine release and NF-kappaB pathway. Br J Pharmacol 148: 478-489.

Hansel NN, Gao L, Rafaels NM, Mathias RA, Neptune ER, Tankersley C, et al. 2009. Leptin receptor polymorphisms and lung function decline in COPD. Eur Respir J 34: 103-110.

Hinson JA, Michael SL, Ault SG, Pumford NR. 2000. Western blot analysis for nitrotyrosine protein adducts in livers of saline-treated and acetaminophen-treated mice. Toxicol Sci 53: 467-473.

Honda Y, Takahashi H, Kuroki Y, Akino T, Abe S. 1996. Decreased contents of surfactant proteins A and D in BAL fluids of healthy smokers. Chest 109: 1006-1009.

Huang KL, Chen CW, Chu SJ, Perng WC, Wu CP. 2008. Systemic inflammation caused by white smoke inhalation in a combat exercise. Chest 133: 722-728.

Kader AK, Liu J, Shao L, Dinney CP, Lin J, Wang Y, et al. 2007. Matrix metalloproteinase polymorphisms are associated with bladder cancer invasiveness. Clin Cancer Res 13: 2614-2620.

Kida K, Oda H, Yamano Y, Kagawa J. 1997. Effects of cigarette smoking on the serum concentration of lung surfactant protein A (SP-A). Eur Respir J 10: 2124-2126.

Kim Y, Lian F, Yeum KJ, Chongviriyaphan N, Choi SW, Russell RM, et al. 2007. The effects of combined antioxidant (beta-carotene, alpha-tocopherol and ascorbic acid) supplementation on antioxidant capacity, DNA single-strand breaks and levels of insulin-like growth factor-1/IGF-binding protein 3 in the ferret model of lung cancer. Int J Cancer 120: 1847-1854.

Klein-Szanto AJ, Iizasa T, Momiki S, Garcia-Palazzo I, Caamano J, Metcalf R, et al. 1992. A tobacco-specific N-nitrosamine or cigarette smoke condensate causes neoplastic transformation of xenotransplanted human bronchial epithelial cells. Proc Natl Acad Sci U S A 89: 6693-6697.

Klinchid J, Chewaskulyoung B, Saeteng S, Lertprasertsuke N, Kasinrerk W, Cressey R. 2009. Effect of combined genetic polymorphisms on lung cancer risk in northern Thai women. Cancer Genet Cytogenet 195: 143-149.

Kourisky R, Happert JL. 1964. Comparisons between bronchopulmonary cancer induced in mice by cigarette smoke tar and human cancer. Bull Assoc Fr Etud Cancer 51: 243-267.

Leikauf GD, Borchers MT, Prows DR, Simpson LG. 2002. Mucin apoprotein expression in COPD. Chest 121: 166S-182S.

Lemjabbar H, Li D, Gallup M, Sidhu S, Drori E, Basbaum C. 2003. Tobacco smoke-induced lung cell proliferation mediated by tumor necrosis factor alpha-converting enzyme and amphiregulin. J Biol Chem 278: 26202-26207.

Levitzky YS, Guo CY, Rong J, Larson MG, Walter RE, Keaney JF, Jr., et al. 2008. Relation of smoking status to a panel of inflammatory markers: the framingham offspring. Atherosclerosis 201: 217-224.

Li PC, Chen WC, Chang LC, Lin SC. 2008. Substance P acts via the neurokinin receptor 1 to elicit bronchoconstriction, oxidative stress, and upregulated ICAM-1 expression after oil smoke exposure. Am J Physiol Lung Cell Mol Physiol 294: L912-L920.

Liu C, Lian F, Smith DE, Russell RM, Wang XD. 2003. Lycopene supplementation inhibits lung squamous metaplasia and induces apoptosis via up-regulating insulin-like growth factor-binding protein 3 in cigarette smoke-exposed ferrets. Cancer Res 63: 3138-3144.

Ma B, Kang MJ, Lee CG, Chapoval S, Liu W, Chen Q, et al. 2005. Role of CCR5 in IFN-gamma-induced and cigarette smoke-induced emphysema. J Clin Invest 115: 3460-3472.

Marwick JA, Stevenson CS, Giddings J, MacNee W, Butler K, Rahman I, et al. 2006. Cigarette smoke disrupts VEGF165-VEGFR-2 receptor signaling complex in rat lungs and patients with COPD: morphological impact of VEGFR-2 inhibition. Am J Physiol Lung Cell Mol Physiol 290: L897-L908.

Mollsten A, Kockum I, Svensson M, Rudberg S, Ugarph-Morawski A, Brismar K, et al. 2008. The effect of polymorphisms in the renin-angiotensin-aldosterone system on diabetic nephropathy risk. J Diabetes Complications 22: 377-383.

Morimoto Y, Tsuda T, Hori H, Yamato H, Ohgami A, Higashi T, et al. 1999. Combined effect of cigarette smoke and mineral fibers on the gene expression of cytokine mRNA. Environ Health Perspect 107: 495-500.

Nakamaru Y, Vuppusetty C, Wada H, Milne JC, Ito M, Rossios C, et al. 2009. A protein deacetylase SIRT1 is a negative regulator of metalloproteinase-9. FASEB J 23: 2810-2819.

Newland N, Richter A. 2008. Agents associated with lung inflammation induce similar responses in NCI-H292 lung epithelial cells. Toxicol In Vitro 22: 1782-1788.

Noordhoek JA, Postma DS, Chong LL, Vos JT, Kauffman HF, Timens W, et al. 2003. Different proliferative capacity of lung fibroblasts obtained from control subjects and patients with emphysema. Exp Lung Res 29: 291-302.

Oltmanns U, Chung KF, Walters M, John M, Mitchell JA. 2005. Cigarette smoke induces IL-8, but inhibits eotaxin and RANTES release from airway smooth muscle. Respir Res 6: 74.

Pignatelli B, Li CQ, Boffetta P, Chen Q, Ahrens W, Nyberg F, et al. 2001. Nitrated and oxidized plasma proteins in smokers and lung cancer patients. Cancer Res 61: 778-784.

Plataki M, Tzortzaki E, Rytila P, Demosthenes M, Koutsopoulos A, Siafakas NM. 2006. Apoptotic mechanisms in the pathogenesis of COPD. Int J Chron Obstruct Pulmon Dis 1: 161-171.

Richter A, O'Donnell RA, Powell RM, Sanders MW, Holgate ST, Djukanovic R, et al. 2002. Autocrine ligands for the epidermal growth factor receptor mediate interleukin-8 release from bronchial epithelial cells in response to cigarette smoke. Am J Respir Cell Mol Biol 27: 85-90.

Robin M, Dong P, Hermans C, Bernard A, Bersten AD, Doyle IR. 2002. Serum levels of CC16, SP-A and SP-B reflect tobacco-smoke exposure in asymptomatic subjects. Eur Respir J 20: 1152-1161.

Rom WN, Travis WD, Brody AR. 1991. Cellular and molecular basis of the asbestos-related diseases. Am Rev Respir Dis 143: 408-422.

Sampsonas F, Kaparianos A, Lykouras D, Karkoulias K, Spiropoulos K. 2007. DNA sequence variations of metalloproteinases: their role in asthma and COPD. Postgrad Med J 83: 244-250.

Schneider J, Presek P, Braun A, Bauer P, Konietzko N, Wiesner B, et al. 1999. p53 protein, EGF receptor, and anti-p53 antibodies in serum from patients with occupationally derived lung cancer. Br J Cancer 80: 1987-1994.

Seno K, Zhu JH, Barrett JD, Eggena P, Scremin OU, Lam K, et al. 1997. Cigarette smoke increases gastric ulcer size in part by an angiotensin II-mediated mechanism in rats. Dig Dis Sci 42: 74-78.

Shao MX, Nakanaga T, Nadel JA. 2004. Cigarette smoke induces MUC5AC mucin overproduction via tumor necrosis factor-alpha-converting enzyme in human airway epithelial (NCI-H292) cells. Am J Physiol Lung Cell Mol Physiol 287: L420-L427.

Stabile LP, Davis AL, Gubish CT, Hopkins TM, Luketich JD, Christie N, et al. 2002. Human non-small cell lung tumors and cells derived from normal lung express both estrogen receptor alpha and beta and show biological responses to estrogen. Cancer Res 62: 2141-2150.

Suzuki M, Betsuyaku T, Nagai K, Fuke S, Nasuhara Y, Kaga K, et al. 2008. Decreased airway expression of vascular endothelial growth factor in cigarette smoke-induced emphysema in mice and COPD patients. Inhal Toxicol 20: 349-359.

Vernooy JH, Drummen NE, van Suylen RJ, Cloots RH, Moller GM, Bracke KR, et al. 2009. Enhanced pulmonary leptin expression in patients with severe COPD and asymptomatic smokers. Thorax 64: 26-32.

Vlahos R, Bozinovski S, Jones JE, Powell J, Gras J, Lilja A, et al. 2006. Differential protease, innate immunity, and NF-kappaB induction profiles during lung inflammation induced by subchronic cigarette smoke exposure in mice. Am J Physiol Lung Cell Mol Physiol 290: L931-L945.

Weitz JI, Silverman EK, Thong B, Campbell EJ. 1992. Plasma levels of elastase-specific fibrinopeptides correlate with proteinase inhibitor phenotype. Evidence for increased elastase activity in subjects with homozygous and heterozygous deficiency of alpha 1-proteinase inhibitor. J Clin Invest 89: 766-773.

Willey JC, Grafstrom RC, Moser CE, Jr., Ozanne C, Sundquvist K, Harris CC. 1987. Biochemical and morphological effects of cigarette smoke condensate and its fractions on normal human bronchial epithelial cells in vitro. Cancer Res 47: 2045-2049.

Wright JL, Tai H, Wang R, Wang X, Churg A. 2007. Cigarette smoke upregulates pulmonary vascular matrix metalloproteinases via TNF-alpha signaling. Am J Physiol Lung Cell Mol Physiol 292: L125-L133.

Zangar RC, Bollinger N, Verma S, Karin NJ, Lu Y. 2008. The nuclear factor-kappa B pathway regulates cytochrome P450 3A4 protein stability. Molecular Pharmacology 73: 1652-1658.

Zayas JG, O'Brien DW, Tai S, Ding J, Lim L, King M. 2004. Adaptation of an amphibian mucociliary clearance model to evaluate early effects of tobacco smoke exposure. Respir Res 5: 9.

Zhang W, Song F, Windsor LJ. 2009. Cigarette smoke condensate affects the collagen-degrading ability of human gingival fibroblasts. J Periodontal Res 44: 704-713.

Zhou H, Alexis NE, Almond M, Donohue J, LaForce C, Bromberg PA, et al. 2009. Influence of C-159T SNP of the CD14 gene promoter on lung function in smokers. Respir Med 103: 1358-1365.
